# Suppressive Effects of Resveratrol Treatment on The Intrinsic Evoked Excitability of CA1 Pyramidal Neurons

**DOI:** 10.22074/cellj.2015.13

**Published:** 2015-10-07

**Authors:** Gholamhossein Meftahi, Zohreh Ghotbedin, Mohammad Javad Eslamizade, Narges Hosseinmardi, Mahyar Janahmadi

**Affiliations:** 1 Neuroscience Research Center and Department of Physiology, Medical School, Shahid Beheshti University of Medical Sciences, Tehran, Iran; 2 Neuroscience Research Center, Baqiyatallah (a.s.) University of Medical Sciences, Tehran, Iran; 3 Department of Biology, Shahid Chamran University, Ahvaz, Iran; 4 Shefa Neuroscience Research Center, Khatam Al Anbia Hospital, Tehran, Iran; 5 Department of Neuroscience, School of Advanced Medical Technology, Iran University of Medical Sciences, Tehran, Iran

**Keywords:** Resveratrol, Electrophysiology, Action Potential, Neurons, Whole Cell Patch Clamp

## Abstract

**Objective:**

Resveratrol, a phytoalexin, has a wide range of desirable biological actions. Despite a growing body of evidence indicating that resveratrol induces changes in neu- ronal function, little effort, if any, has been made to investigate the cellular effect of res- veratrol treatment on intrinsic neuronal properties.

**Materials and Methods:**

This experimental study was performed to examine the acute effects of resveratrol (100 µM) on the intrinsic evoked responses of rat Cornu Ammonis (CA1) pyramidal neurons in brain slices, using whole cell patch clamp re- cording under current clamp conditions.

**Results:**

Findings showed that resveratrol treatment caused dramatic changes in evoked responses of pyramidal neurons. Its treatment induced a significant (P<0.05) increase in the after hyperpolarization amplitude of the first evoked action potential. Resveratrol-treated cells displayed a significantly broader action potential (AP) when compared with either control or vehicle-treated groups. In addition, the mean instantaneous firing frequency between the first two action potentials was significantly lower in resveratrol-treated neurons. It also caused a significant reduction in the time to maximum decay of AP. The rheobase current and the utilization time were both significantly greater following resveratrol treatment. Neurons exhibited a significantly depolarized voltage threshold when exposed to resveratrol.

**Conclusion:**

Results provide direct electrophysiological evidence for the inhibitory effects of resveratrol on pyramidal neurons, at least in part, by reducing the evoked neural activity.

## Introduction

In recent years, many naturally occurring compounds consumed by human populations have gained considerable attention for the treatment of neurodegenerative diseases. In this context, resveratrol (3, 5, 4-trihydroxy-trans-stilbene), a phytoalexin produced by a variety of plant species including grapes, mulberries, cranberries and peanuts has been shown to exert a wide range of actions, including neuroprotective effects (1,4). In addition, it has been reported that resveratrol may have a potential role in the control of heart disease, atherosclerosis, arthritis, anti-aging, and autoimmune disorders (5,7). 

It has also been reported that it may reduce excite toxicity via an inhibitory effect on postsynaptic glutamatergic transmission and by enhancing glutamate uptake in astrocytes following oxidative stress (8,10). Improvement of cognitive function by resveratrol treatment has been attributed to increasing insulin-like growth factor-I (IGF-I) production and promoting angiogenesis and neurogenesis in the hippocampal astrocytes through stimulation of sensory neurons in the gastrointestinal tract. It could also delay the onset of neurodegenerative disease and prevent learning impairment in transgenic Alzheimer’s disease models (11,14). 

In addition, resveratrol has a large attenuation effect on the increased spontaneous excitatory post synaptic currents (sEPSCs) in hippocampal pyramidal neurons (15). Chronic treatment with resveratrol could intensively inhibit the release of extracellular glutamate and aspartate during ischemia/reperfusion. Therefore, the neuroprotective effects of resveratrol may be partly due to its ability to attenuate extracellular excitotoxic glutamate and aspartate accumulation (16). The inhibitory effect of resveratrol on voltage-activated K ^+^currents in rat hippocampal neurons has also been suggested to be useful in treating ischemic brain injury (17). The antinociceptive action of resveratrol in the formalin test has been found to be mediated by opening the Ca ^2+^activated-K ^+^channels (18). In rat dorsal root ganglion, resveratrol suppressed Na ^+^currents in a concentration-dependent manner (19). Its application can also reduce the frequency of seizures and improve the pathological damage to hippocampal Cornu Ammonis (CA) CA1 and CA3 pyramidal neurons after kainic acid-induced temporal lobe seizures (20). 

These observations provide evidence that resveratrol can be applicable in protecting some animals suffering from different types of neurological disorders. However, despite extensive studies on the biological effects of resveratrol, little is known about its cellular mechanisms, including the actual electrophysiological actions on intrinsic neuronal evoked activity. 

Considering the proved neuroprotective properties of resveratrol, therefore the main aim of this study was to determine the effects of resveratrol on evoked electrophysiological responses of CA1 pyramidal neurons. 

## Materials and Methods

This experimental study was performed in accordance with the Ethical guidelines of Shahid Beheshti University of Medical Sciences and efforts were made to minimize animal suffering and the number of animals used. A total of 13 young adult male Wistar rats ( 4-6 weeks old were used and divided into 3 groups: control (n=4 and 10 cells), vehicle [dimethyl sulfoxide (DMSO)]-treated (n=4 and 8 cells) and resveratrol-treated (n=5 and 12 cells). They were kept at a room temperature of 23 ± 3˚C with a 12:12 hour light/dark cycle. Rats were given free access to rodent chow and tap water. 

### Hippocampal slice preparation and whole cell patch clamp recording

Whole cell patch clamp recording was performed as previously described (21,22). Briefly, animals were anesthetized via inhalation of ether. They were then decapitated and their brains rapidly removed and placed in ice-cold artificial-spinal fluid (ACSF) containing (in mM) 206 sucrose, 2.8 KCl, 1 CaCl _2_, 1 MgCl _2_, 2 MgSO _4_, 1.25 NaH _2_PO _4_, 26 NaHCO ^3^, 10 D-glucose, and equilibrated to a pH=7.4 (with 95% O_2_and 5% CO ^2^); the osmolarity was adjusted to 295 mOsm. All salts were purchased from Sigma (UK). The hippocampus then was dissected out of the brain, and 300 µm thick transverse slices were obtained using a vibrating microtome (752 M, Campden Instruments Ltd., UK) before being transferred to an incubating chamber containing oxygenated ACSF (in mM): 2 MgSO _4_, 2.8 KCl, 2 CaCl _2_, 124 NaCl, 1.25 NaH_2_ PO _4_, 26 NaHCO _3_, 10 D-glucose, pH=7.4, 295 mOsm, for at least 30 minutes at 32-35˚C. After incubation, slices were kept at room temperature (23-25˚C) until individually transferred into the recording chamber. 

### Intracellular whole-cell current-clamp recordings

Hippocampal slices were transferred to a submerged recording chamber on the stage of an upright microscope (Olympus, BX 51WI, Japan). CA1 pyramidal neurons were then visualized with a 60×water immersion objective using Nomarskitype differential interference contrast imaging with infrared illumination. Images were captured with a CCDcamera (Hmamatsu, ORSA, Japan). The slices were continuously superfused with normal Resveratrol At tenuates Neuronal Exci tabili ty oxygenated ACSF at room temperature (23-25˚C). Whole cell patch clamp recordings were obtained from hippocampal CA1 pyramidal neurons using Multiclamp 700 B amplifier, Digidata 1320 A/D and pClamp 9.2 software (Axon Instruments, Molecular Devices Co., Sunnyvale, CA). Electrophysiological responses were filtered at 5 kHz and sampled at 10 kHz and stored on a personal computer for offline analysis. Patch pipettes were pulled with a vertical microelectrode puller (PC10, Narishige, Tokyo, Japan) and had resistance of 4-7 MΩ when filled with internal solution containing (in mM): 135 potassium methylsulfate (KMeSO _4_), 10 KCl, 10 Hepes, 1 MgCl _2_, 2 Na _2_ATP, and 0.4 Na _2_GTP. The pH of the internal solution was set to 7.3 by KOH and osmolarity was adjusted to 290 mOsm. All salts were purchased from Sigma, UK. 

To investigate the effect of resveratrol on the electrically evoked responses, action potentials were elicited applying depolarizing current pulses (520 mseconds) ranging from 100-500 pA in 100 pA increments, in the presence of synaptic channel blockers (100 µM picrotoxin and 1 mM kynurenic acid, Sigma, UK). Alterations in evoked excitability was also examined using a depolarizing ramp current (880 mseconds) with a slope of 3.1 pA/ms from 0 pA to 220 pA. 

The following electrophysiological parameters were measured and assessed. The after hyperpolarization potential (AHP) amplitude was measured as the difference between the spike threshold and the minimum voltage following the AP peak. Action potential half width was measured at half amplitude of AP. Instantaneous firing frequency (1/interspike interval) was calculated from a train of AP evoked by pulses of 625 mseconds duration. Rheobase current and utilization time were defined as the minimum current threshold required to evoke AP and the first spike latency during a ramp depolarizing current, respectively. 

### Drug application

Resveratrol (Tocris, Bristol, UK) was dissolved in DMSO, to prepare a stock solution and was diluted to final concentration of 100 µM (9,15,19). The final concentration of DMSO was less than 0.1%. The recording chamber with a volume of ~1.5 ml was continuously superfused with oxygenated ACSF at 1.5-2 ml/minutes. Following the 8 minutes perfusion with ACSF containing resveratrol whole cell patch clamp recordings were done under current clamp conditions. The entire recording period for each cell lasted approximately 25 minutes. 

### Cell selection criteria

Patched CA1 pyramidal neurons were accepted for study if they had a patch seal resistance of greater than 1GΩ, a series resistance less than 20% of membrane resistance and a resting membrane potential between -55 to -65 mV. 

### Data analysis

The results obtained were expressed as mean ± standard error of mean (SEM). Statistical significance was assessed by one-way ANOVA followed by Tukey’s post hoc test, using SPSS software (version 16). Values of P≤0.05 were considered to be significant. 

## Results

**Fig.1:**
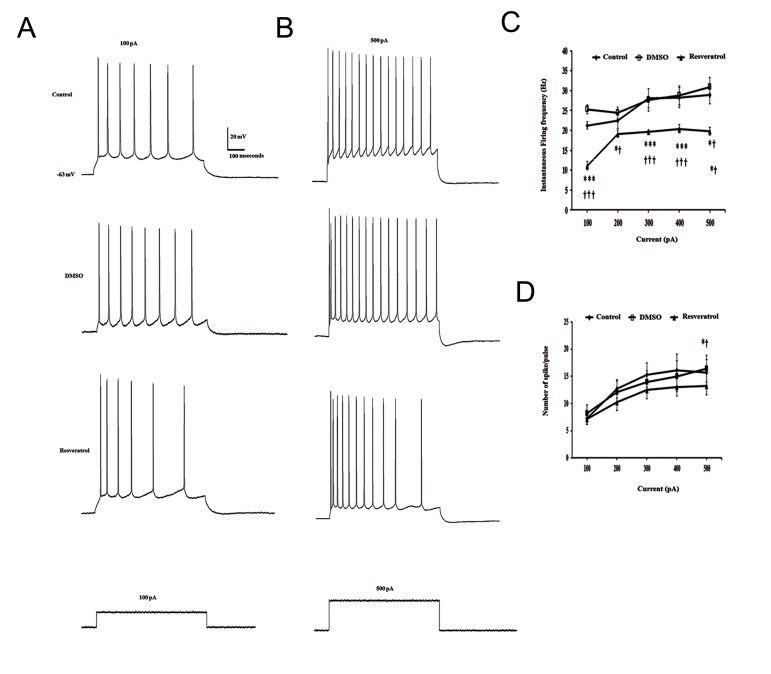
Suppressive effect of resveratrol on the evoked responses of CA1 pyramidal neurons representative membrane voltage responses to depolarizing current pulses of A. 100 pA, B. 500 pA before and after resveratrol treatment, C. The effect of resveratrol on the intrinsic firing frequency and D. The number of evoked spike per pulse. CA; Cornu ammonis and DMSO; Dimethyl sulfoxide .

The effects of resveratrol (100 μM) on intrinsic evoked excitability were assessed after blocking synaptic activities. Under current clamp conditions, depolarization of CA1 pyramidal neurons by injecting a series of current pulses with increasing amplitude led to a significant decrease in the instantaneous firing frequency in resveratrol-treated neurons when compared to either control or DMSO-treated neurons [F(2,15)=55.82, P<0.001; F(2,18)=8.41, P<0.05; F(2,18)=28.74, P<0.001; F(2,18)=17.36, P<0.001; F(2,14)=10.75, P<0.01, in response to 100 to 500 pA depolarizing current steps, respectively, Fig.1A-C]. In the presence of resveratrol, the number of evoked AP was also significantly decreased in response to a 500 pA depolarizing current injection compared to control and vehicle treated groups ( One way ANOVA, F(2,20)=3.68, P<0.05, Fig.1D). 

**Fig.2:**
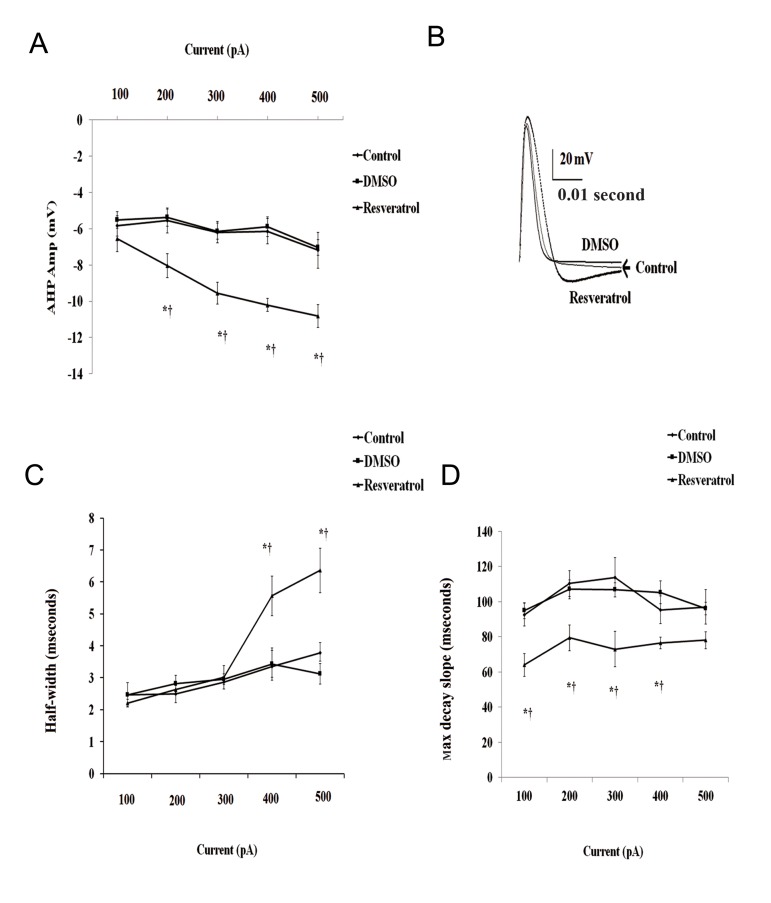
Effect of resveratrol on evoked action potential parameters. A. Histograms show the mean amplitude of after hyperpolarization potential (AHP). Superimposed traces of first evoked AP in response to 500 pA depolarizing current pulse recorded from control, dimethyl sulfoxide (DMSO)-treated and resveratrol treated neurons is shown in B. C. The mean half-width of action potential (AP) and D. The average time to maximum decay slope. *; Represents significant difference between control and resveratrol-treated groups (P<0.05) and †; Donates significant difference DMSO and resveratrol-treated groups (P<0.05).

In addition, comparison of the evoked action potentials before and after application of resveratrol revealed a significantly larger AHP amplitude ( One way ANOVA, F(2,17)=16.34, F(2,18)=18.98), F(2,19)=34.57 and F(2,18)=24.93, in response to 200, 300, 400 and 500 pA, respectively, P<0.001, Fig.2A, B). Resveratrol treatment also caused a significant prolongation of action potential duration at 50% repolarization in response to higher depolarizing current injections ( One way ANOVA, F(2,16)=5.86 and F(2,14)=10.47, in response to 400 pA and 500 pA current injections, respectively, Fig.2B, C). 

535 After the application of 100 μM resveratrol to the bath, depolarizing current injections resulted in a significant reduction in the time to max decayslope of AP ( One way ANOVA, F(2,11)=82.67, F(2,13)=38.78, F(2,13)=62.28, F(2,13)=66.55, F(2,11)=34.68, P<0.001, Fig.2C). 

**Fig.3:**
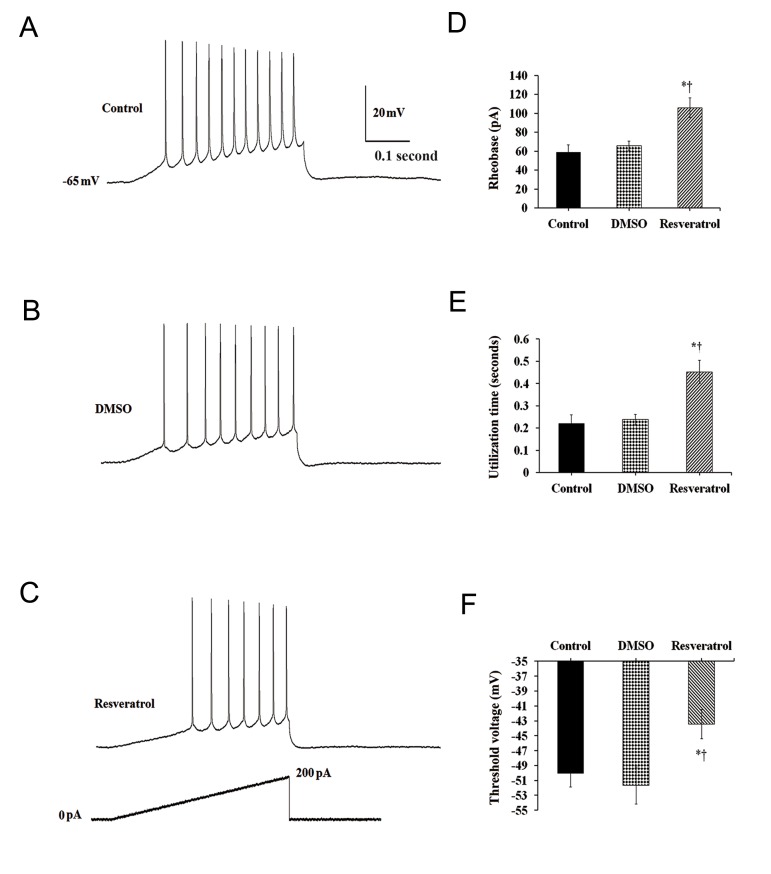
Effect of resveratrol on neuronal excitability. A. Representative voltage responses to a depolarizing current ramp in control, B. Dimethyl sulfoxide (DMSO) -treated and C. Resveratroltreated groups, D. Histograms summarizing the effect of resveratrol on the rheobase current), utilization (latency to the first spike) and E. Voltage threshold compared to control and vehicle-treated neurons. *; Represents significant difference between control and resveratroltreated groups (P<0.05) and †; Donates significant difference DMSO and resveratrol-treated groups (P<0.05).

To further assess alteration of intrinsic neuronal excitability after resveratrol application, rheobase (threshold current), action potential voltage threshold and utilization time were measured in response to a depolarizing current ramp from 0 pA to 220 pA lasting 880 mseconds. (Fig .3A). 

The average rheobase of CA1 pyramidal neurons was significantly higher after resveratrol treatment when compared to control and vehicletreated groups ( One way ANOVA, F(2,18)= 8.56, P<0.01, Fig.3B). 

Addition of resveratrol to the ACSF led to a significant increase in the utilization time (0.451 ± 0.05 seconds) compared to control (0.220 ± 0.03 seconds) and DMSO (0.238 ± 0.023 seconds) groups (One way ANOVA, F(2,20)=9.62, P<0.01, Fig.3C). The average AP voltage threshold was -43.45 ± 1.96 mV (P<0.05), after resveratrol application when compared to control (50.07 ± 1.79 mV) and DMSO-treated (51.67 ± 2.48 mV) groups, respectively (One way ANOVA, F(2,19)=4.58, Fig.3D). There were no significant difference in the measured electrophysiological parameters between control and vehicle-treated neurons.

## Discussion

This study set out with the aim of assessing the acute electrophysiological effects of resveratrol on CA1 hippocampal pyramidal neurons. Here, the intrinsic evoked ﬁring properties of CA1 pyramidal neurons were investigated for the first time after blocking fast synaptic inputs. Several studies have provided evidence that resveratrol exerts neuroprotective activities (23-25). However, very little is known about its effects on evoked electrophysiological properties.

The ﬁndings of the present study revealed that extracellular application of resveratrol profoundly affected the evoked electrophysiological response properties of CA1 pyramidal neurons.

Resveratrol treatment produced a broader action potential width and larger AHP amplitude which was associated with a lower neuronal excitability. The enhancement of the AHP amplitude could be due to the larger Ca^2+^ entry allowed by the wider AP (26). It has been shown that the AHP is an intrinsic property of hippocampal pyramidal neurons and is caused by the contribution of Ca^2+^-dependent K+ current. There has been an increasing accumulation of evidence suggesting that resveratrol modulates the activities of several types of ion channels, such as stimulation of Ca^2+^- activated K (K_Ca2+_) channels in vascular endothelial cells, inhibition of L-type Ca^2+^ currents in ventricular myocytes (15, 27), and inhibition of Na^+^ channel currents in rat dorsal root ganglion neurons (19). It has been shown that the stimulatory effect of resveratrol on K_Ca2+_ was associated with membrane hyperpolarization (28), which thereby may cause enhancement of AHP amplitude. Here, the large AHP amplitude observed in resveratroltreated neurons could be due to the activation of K_Ca2+_ which was accompanied with a significant decrease in firing frequency.

The suppressive effect of resveratrol on neuronal excitability observed in the present study is consistent with those of Li et al. (16, 29) who found that resveratrol inhibited the spontaneous discharge and ischemia-induced glutamate release in the rat hippocampal CA1 region. The present result is also consistent with Li et al. (28) who found resveratrol can significantly reduce epileptiform discharges induced by L-glutamate and reverse the increased discharges induced by Bay K8644 which strongly suggests the inhibitory effects of resveratrol on voltage-gated L-type calcium channels. Suppression of voltage-dependent Ca^2+^ channel activity in rat cerebrocortical nerve terminals as a potential mechanism underlying the inhibition of glutamate release by resveratrol has also been reported (30). This also could be the reason for decreasing the neuronal excitability caused by resveratrol.

Acute exposures of CA1 pyramidal neurons to resveratrol also resulted in a significant increase in the rheobase current and utilization time and a significant depolarized threshold voltage, which all contribute to the decreased neuronal excitability. Several factors including action potential threshold and waveform may affect neuronal excitability (31). Modulation of voltage-gated Na^+^ channels regulates the depolarizing rising phase of action potential and firing frequency (32, 33). One possible explanation for the higher rheobase (threshold) current and utilization time, and depolarized threshold voltage following resveratrol treatment could be due to a significant increase in the AHP amplitude, which in turn may lead to fewer Na^+^ channels available for activation. The mechanism for the inhibitory effect of resveratrol on Na^+^ channels reported by Kim et al. (19) could also be the reason for the higher rheobase (threshold) current which reflects the lower excitability seen in neurons treated with resveratrol in the present work.

## Conclusion

The present results further characterized the electrophysiological consequences of the suppressive effects of resveratrol on intrinsic neuronal excitability and electrical responsiveness in CA1 pyramidal neurons. This suppressive action of resveratrol on intrinsic neuronal activity may be one of the underlying mechanisms for its neuroprotective effect against brain diseases which are associated with neuronal hyperexcitability.
